# Fecal and Mucosal Microbiota Profiling in Irritable Bowel Syndrome and Inflammatory Bowel Disease

**DOI:** 10.3389/fmicb.2019.01655

**Published:** 2019-07-17

**Authors:** Alessandra Lo Presti, Francesca Zorzi, Federica Del Chierico, Annamaria Altomare, Silvia Cocca, Alessandra Avola, Fabiola De Biasio, Alessandra Russo, Eleonora Cella, Sofia Reddel, Emma Calabrese, Livia Biancone, Giovanni Monteleone, Michele Cicala, Silvia Angeletti, Massimo Ciccozzi, Lorenza Putignani, Michele Pier Luca Guarino

**Affiliations:** ^1^Department of Infectious Diseases, Istituto Superiore di Sanità, Rome, Italy; ^2^Gastrointestinal Unit, Department of Systems Medicine, University of Rome Tor Vergata, Rome, Italy; ^3^Human Microbiome Unit, Bambino Gesù Children’s Hospital, IRCCS, Rome, Italy; ^4^Unit of Digestive Disease, Campus Bio-Medico University, Rome, Italy; ^5^Unit of Medical Statistics and Molecular Epidemiology, Campus Bio-Medico University, Rome, Italy; ^6^Unit of Clinical Laboratory Science, Campus Bio-Medico University, Rome, Italy; ^7^Human Microbiome Unit and Parasitology Unit, Bambino Gesù Children’s Hospital, IRCCS, Rome, Italy

**Keywords:** gut microbiota, irritable bowel syndrome, inflammatory bowel disease, dysbiosis, inflammation

## Abstract

An imbalance in the bacterial species resulting in the loss of intestinal homeostasis has been described in inflammatory bowel diseases (IBD) and irritable bowel syndrome (IBS). In this prospective study, we investigated whether IBD and IBS patients exhibit specific changes in richness and distribution of fecal and mucosal-associated microbiota. Additionally, we assessed potential 16S rRNA gene amplicons biomarkers for IBD, IBS, and controls (CTRLs) by comparison of taxonomic composition. The relative abundance of bacteria, at phylum and genus/species levels, and the bacterial diversity were determined through 16S rRNA sequence-based fecal and mucosal microbiota analysis. Linear discriminant analysis effect size (LEfSe) was used for biomarker discovery associated to IBD and IBS as compared to CTRLs. In fecal and mucosal samples, the microbiota richness was characterized by a microbial diversity reduction, going from CTRLs to IBS to IBD. β-diversity analysis showed a clear separation between IBD and CTRLs and between IBD and IBS with no significant separation between IBS and CTRLs. β-diversity showed a clear separation between mucosa and stool samples in all the groups. In IBD, there was no difference between inflamed and not inflamed mucosa. Based upon the LEfSe data, the *Anaerostipes* and Ruminococcaceae were identified as the most differentially abundant bacterial taxa in CTRLs. Erysipelotrichi was identified as potential biomarker for IBS, while Gammaproteobacteria, *Enterococcus*, and Enterococcaceae for IBD. This study provides an overview of the alterations of microbiota and may aid in identifying potential 16S rRNA gene amplicons mucosal biomarkers for IBD and IBS.

## Introduction

In humans, more than 100 trillion microorganisms colonize the gastrointestinal tract establishing mutualistic relationships with the host (Haque and Haque, 2017). Metagenomic data indicate that gram-negative bacteroidetes (17–60%) and gram positive firmicutes (35–80%) are the most predominant phyla in healthy individuals (Bäckhed et al., 2005; Cho et al., 2012). Changes in the bacterial species, the so-called dysbiosis, resulting in the loss of intestinal homeostasis, have been described in different intestinal disorders, including Inflammatory Bowel Diseases (IBD) and Irritable Bowel Syndrome (IBS) (Nishida et al., 2018; Rodiño-Janeiro et al., 2018). IBD, consisting of ulcerative colitis (UC), and Crohn’s disease (CD), are chronic, relapsing-remitting, gastrointestinal inflammatory disease which associate with various degrees of intestinal damage, and can promote development of local and extra-intestinal complications (Xavier and Podolsky, 2007). The incidence and prevalence of IBD are highest in westernized nations, with reported hightest prevalence values in Europe of 322 for CD and 505 for UC per 100,000 persons (Molodecky et al., 2012; Ng et al., 2018). The prevalence of IBD exceeded 0.3% in North America, Oceania, and many countries in Europe. The changing nature of IBD, including relapsing and remitting stages, along with potential disease complications can also lead to psychological symptoms of anxiety, and depression (Ng et al., 2018). Although the pathogenesis of IBD is not fully understood, several lines of evidence support the hypothesis that IBD occur in genetically susceptible subjects as a result of an abnormal immune response to autologous bacterial flora following exposure to multiple environmental factors (Strober et al., 2007; Hold et al., 2014). It has also been hypothesized that a breakdown in the balance between putative protective species and “harmful” species could contribute to IBD pathogenesis (Kamada et al., 2013). For instance, many studies have documented reduced bacterial diversity and richness in IBD patients, largely due to decrease of firmicutes and increase of Bacteroidetes phyla (Manichanh et al., 2006, 2012; Willing et al., 2010; Ni et al., 2017).

Irritable bowel syndrome is one of the most common functional gastrointestinal disorders worldwide. Global prevalence, based on Rome III criteria, was estimated at 10–15% (Longstreth et al., 2006). Recent studies using the more restrictive Rome IV criteria (Lacy et al., 2016; Lacy and Patel, 2017), point to lower prevalence rates of 5–6% (Jossan et al., 2017; Van den Houte et al., 2019).

Irritable bowel syndrome is characterized by abdominal pain or discomfort, bloating, and altered bowel habits. Increasing evidence suggests an important role of the intestinal microbiota in the pathophysiology of IBS (Jeffery et al., 2012; Labus et al., 2017; Menees and Chey, 2018). Support for this comes from the observation that IBS can develop after intestinal infection and efficacy of probiotics in the management of IBS patients (Dupont, 2014; Ghoshal and Srivastava, 2014).

To understand the interactions between microbiota, metabolic processes, and pathophysiology it is important to elucidate specific microbial signatures of IBS and IBD.

The aim of this study was to investigate the differences in fecal and mucosal-associated microbiota richness and composition among IBD, IBS patients along with healthy controls (CTRLs), to better define if each disorder have its own microbiota signature. An additional aim of this study was to evaluate potential 16S rRNA gene amplicons biomarkers for IBD and IBS by comparison of taxonomic composition, allowing to predict the bacteria that concisely differentiate among the groups being compared, or to identify the alterations shared.

## Materials and Methods

This prospective, multicenter study was conducted on patients with diagnosis of IBD or IBS compared to healthy subjects (CTRLs), consecutively enrolled at the Gastroenterology Unit of the Tor Vergata Hospital and the Department of Gastroenterology of Campus Biomedico University of Rome between 2015 and 2017.

### Study Population

A complete demographic and clinical evaluation of patients and CTRLs was performed by a Gastroenterologist during the first visit of enrollment.

Below the inclusion and exclusion criteria for patients and CTRLs:

(a) IBD patients:
  Inclusion criteria: (1) Diagnosis of IBD for at least 3 months according to standard Montreal classification (Satsangi et al., 2006). (2) Patients with IBD with colic or ileocolic localization. (3) Patients aged 25–60 years.
  Exclusion criteria: (1) Use of antibiotics or any other probiotic bacterial supplement in the previous 3 months. (2) Use of non-steroidal anti-inflammatory drugs (NSAIDs) in the previous 3 months. (3) Reported recent diagnosis (less than 3 months) of bacterial or parasitic infections of the gastrointestinal tract.
(b) IBS patients:
  Inclusion criteria: diagnosis of IBS performed by using the following diagnostic-therapeutic procedures: clinical evaluation and blood/stool test; questionnaire of intestinal functional disorders, elaborated according to the Rome IV criteria (Lacy et al., 2016; Lacy and Patel, 2017); colonoscopy (RSCS) with multiple biopsies.
  Exclusion criteria: (1) Use of antibiotics or any other probiotic bacterial supplement in the previous 3 months. (2) Use of NSAIDs in the previous 3 months. (3) Reported recent diagnosis (less than 3 months) of bacterial or parasitic infections of the gastrointestinal tract. (4) Severe psychiatric disease as the dominant clinical problem. (5) Other severe diseases, and a history of drug or alcohol abuse.
(c) CTRLs
  Inclusion criteria: (1) Gastrointestinal asymptomatic subjects (using a questionnaire to exclude chronic diseases and any current gastrointestinal symptoms). (2) Up to 60 years of age who undergo colonoscopy for colorectal cancer screening. (3) Absence of macroscopic lesions (including the presence of *diverticulae*). (4) Absence of microscopic lesions evident on the histological examination of colonic biopsy samples taken during the colonoscopy. The CTRLs exclusion criteria were the same described for IBS.

### Study Protocol and Sample Collections

At the baseline visit all the enrolled patients underwent endoscopic examination of the lower digestive tract conducted to explore the cecum, after preparation with polyethylene glycol (PEG) (4 l) and a low fiber diet 3 days prior to endoscopy. Mucosal biopsies were collected from sigmoid colon in all of patients and CTRLs for the routine histological examinations and for the microbiome assessment. In IBD patients, in relation to disease localization, we collected biopsies from the injured mucosa for routine histological examinations and microbiome assessment.

Moreover, when applicable, only for microbiome assessment, in IBD patients was collected an additional biopsy from macroscopic healthy mucosa, by sampling the healthy upstream colon segment. All patients collected a stool sample the day before the preparation with PEG. All biopsies and fecal samples were immediately stored at −80°C, until processing to strictly prevent anaerobic bacteria from being exposed to oxygen and to avoid bacterial overgrowth before DNA extraction.

### DNA Extraction, Amplification for Pyrosequencing, Statistical Analysis

All mucosal and fecal samples were submitted to DNA extraction. DNA from mucosal samples (approximately 1 mm × 2 mm) was automatically extracted by the EZ1 biorobot using EZ1 DNA tissue kit following manufacturer’s instructions (Qiagen, Germany). Fecal DNA was manually extracted, starting from 200 mg of feces, by the QIAamp DNA Stool Mini Kit (Qiagen, Germany). The V1-V3 regions (520 bp) of the 16S ribosomal RNA locus were amplified for the next pyrosequencing step on a 454- Junior Genome Sequencer (Roche 454 Life Sciences, Branford, CT, United States), according to the pipeline described in Ercolini et al. (2012) and Ercolini et al. (2012). Primers were barcoded by 8 unique nucleotide sequences (Roche 454 Life Sciences, Branford, CT, United States). The polymerase chain reactions were performed, starting from 0.5 ng of DNA, using a Hi-Fi PCR Taq polymerase (FastStart^TM^ High Fidelity PCR System, dNTPack, Roche Diagnostics, Mannheim, Germany), guaranteeing high specificity, sensitivity and accuracy during PCR amplification. Amplicon DNA were quantified by Quant-iT PicoGreen dsDNA kit (Life Technologies Corporation, Oregon, United States) following the manufacturer’s instructions, and then pooled in equal concentrations, prior the sequencing reactions. The 454 amplicon signal processing was applied to subtract background and normalize the images process and to transform the captured images into read flowgrams and basecalls with associated per-base quality scores (GS sequencer software v. 2.7, Roche Diagnostics, Mannheim, Germany). The pre-processed reads was trimmed on the base of ends signal quality and to exclude and the adaptor sequences (GS sequencer software v. 2.7, Roche Diagnostics). Raw sequences, obtained from each single sample, were analyzed by using QIIME 1.9.0 software (Caporaso et al., 2010). In order to guarantee a higher level of accuracy in terms of operational taxonomic units (OTUs) detection, after demultiplexing, reads with an average quality score lower than 25, shorter than 300 bp, and with an ambiguous base calling were excluded from the analysis. Sequences that passed the quality filter were denoised (Reeder and Knight, 2010) and singletons were excluded. The denoised sequences were chimera-checked by *identify_chimeric_seqs.py* using both Blast_fragments and ChimeraSlayer^1^ approaches. The OTUs defined by a 97% of similarity were *de novo* picked (*pick_de_novo_otus.py*) and the representative sequences were submitted to PyNAST for the sequence alignment (Caporaso et al., 2010), and to UCLUST for sequence clustering (Edgar, 2010). The database for OTUs matching was greengenes (v 13.8). This script produces an OTU mapping file (*pick_otus.py*), a representative set of sequences (*pick_rep_set.py*), a sequence alignment file (*align_seqs.py*), taxonomy assignment file (*assign_taxonomy.py*), a filtered sequence alignment (*filter_alignment.py*), a phylogenetic tree (*make_phylogeny.py*) and a biom-formatted OTU table (*make_otu_table.py*). After rarefying (rarefaction sequences counts: 2870 sequences for stool samples and 980 sequences for tissue samples), the alpha diversity analysis was performed for both fecal and biopsy sample groups. The β-diversity tests by unweighted and weighted UniFrac metrics were carried out by QIIME software using *beta_diversity_through_plots.py* and plotted by PCoA plot; PERMANOVA test with 999 permutations was applied to unweight and weighted UniFrac distance matrices (*compare_categories.py*); the *group_significance.py* script was used to perform Kruskal-Wallis test to compare OTU frequencies across samples (Navas-Molina et al., 2013). Taxonomic levels phylum and genus/species were studied, and raw *p* value <0.05 and false discovery rate adjusted P (pFDR) < 0.05 were considered as statistically significant.

All sequencing data associated with this study were uploaded to the NCBI bioproject database: PRJNA391149^2^.

### Comparison of Taxonomic Composition According to Disease Status by LEfSe for 16S rRNA-Based Metagenomic Biomarker

Linear discriminant analysis effect size (LEfSe) (Segata et al., 2011), an algorithm used to discover high-dimensional biomarkers characterizing the differences between biological conditions, to identify taxa that differed consistently between sample types, was used for 16S rRNA gene amplicons biomarker discovery associated to IBD and IBS compared to CTRLs on biopsy specimens.

Linear discriminant analysis effect size employs the non-parametric factorial Kruskal-Wallis sum-rank test (α = 0.05) to identify taxa with significantly different abundances between categories, followed by LDA to estimate the effect size of each feature of the differential abundance. The differences in abundance were regarded as statistically significant when the logarithmic LDA score was >2.0. If multiple varieties with different ranks showed significance in the same taxon, the lowest-ranked varieties were regarded as responsible.

## Results

### Study Population

A total of 129 individuals were recruited in this study as part of the research project (code: WFR- GR-2011-02350817) financed by the Ministry of Health (Italy). Specifically, 38 (29.5%) IBD patients from the Department of Medicine and Gastroenterology of Tor Vergata Hospital, 44 (34.1%) IBS patients, and 47 (36.4%) CTRL subjects from the Gastroenterology Unit of the Campus Bio-Medico Hospital (Rome, Italy) were enrolled from 2015 to 2017.

The median age of the study population was 51 years (44–56) (p25 and p75, respectively). Males represented 48% of the study population. The demographic and clinical characteristics of IBD and IBS patients and of CTRLs are shown in Tables 1, 2. The IBS population showed a predominance of female patients, compared to IBD and CTRLs, and due to the prevalence of the disease.

**TABLE 1:** The demographic and clinical characteristics of IBD patients.

**IBD Patient Characteristics**	**IBD**
	**(*N* = 38)**
Crohn’s disease	7
Ulcerative colitis	31
Age, years median (range)	48 (21–74)
Sex, male N (%)	21 (55.2%)
**Smoking habits, N (%)**	
Yes	2 (5.3%)
No	36 (94.7%)
**Disease activity in UC patients, N (%)^a^**	
Remission	3 (9.6%)
Mild	7 (22.6%)
Moderate	11 (35.6%)
Severe	10 (32.2%)
**Disease activity in CD patients, N (%)^b^**	
Remission	2 (28.6%)
Mild	0
Moderate	3 (43.8%)
Severe	2 (28.6%)
**CD behavior, N (%)**	
B1: Inflammatory	2 (28.6%)
B2: Stricture	5 (71.4%)
B3: Penetrating	0
**CD location, N (%)**	
L1: Ileal	5 (71.4%)
L2: Colonic	1 (14.3%)
L3: Ileocolonic	1 (14.3%)
**UC location, N (%)**	
E1: Proctitis	2 (6.4%)
E2: Left-sided	14 (45.2%)
E3: Extensive	15 (48.4%)
**Endoscopist assessment of severity, N (%)^c^**	
Mild	8 (21.1%)
Moderate	13 (34.2%)
Severe	17 (44.7%)
**Previus surgery, N (%)**	
CD: Ileo colonic resection	3 (42%)
UC	0
**Concomitant medication, N (%)**	
5-aminosalicylic acid or sulfasalazine	20 (51.6%)
TNFs alone	3 (7.8%)
Thiopurine alone	2 (5.3%)
Steroids alone	11 (28.9%)
Steroids plus anti-TNF	2 (6.4%)

*a, adapted from Truelove & Witts; b, adapted from Harvey-Bradshaw Index; c, adapted from Mayo endoscopic score for UC patients or simple endoscopic score for CD patients; NA, not applicable.*

**TABLE 2:** The demographic and clinic characteristics of IBS patients and control subjects.

**Subject characteristics**		**IBS (*N* = 44)**	**CTRLs (*N* = 47)**
	**Age, years median (range)**	**48 (28–59)**	**54 (50–59)**

	**BMI (mean)**	**24**	**23**
Sex, N (%)	Male	14 (32)	27 (57)
	Female	30 (68)	20 (43)
Predominant bowel habit, N (%)	Diarrhea	16 (36)	NA
	Constipation	18 (41)	NA
	Alternating	10 (23)	NA
Concomitant therapies, N (%)	Antispasmodics	7 (16)	0
	Antidepressant	3 (7)	1 (2)
	Laxatives	8 (18)	0

*NA, not applicable.*

### Fecal and Mucosal Sample Collections

A total of 107 fecal samples were included in this study and processed, specifically 30 from IBD patients, 36 from IBS patients, and 41 from CTRLs (22 subjects did not collect fecal samples).

A total of 142 biopsy specimens were obtained from 126 subjects. Specifically, 45 biopsies from CTRLs, 44 from IBS patients and 53 from IBD patients (37 from inflamed intestinal areas, and 16 from not inflamed areas) were collected.

### Fecal Microbiota Composition and Distribution

A total of 307,036.00 sequencing reads were obtained from the 107 fecal samples. The differences of microbiota in IBD, IBS, and CTRLs were measured by the α- and β-diversities. In stool samples, the microbiota richness, based on the Shannon and Chao I indexes, was characterized by a diversity reduction going from CTRLs to IBS to IBD (Table 3).

**TABLE 3:** Fisher’s least significant difference (LSD) test on α-diversity indexes.

**Stool**			**Biopsy**		
**Stool**	**Mean**	***p* value**	**Biopsy**	**Mean**	***p* value**
**Shannon index**					
CTRL	**5.14**	**0**.**000**	CTRL	**4.25**	**0**.**000**
IBD	**4.06**		IBD	**3.45**	
CTRL	5.14	0.284	CTRL	4.25	0.649
IBS	4.92		IBS	4.16	
IBD	**4.06**	**0**.**000**	IBD	**3.45**	**0**.**017**
IBS	**4.92**		IBS	**4.16**	
			IBD I^*^	3.05	0.878
			IBD NI^*^	3.16	
**Chao1 index**					
CTRL	**365.05**	**0**.**012**	CTRL	**156.29**	**0**.**001**
IBD	**244.56**		IBD	**98.67**	
CTRL	365.05	0.108	CTRL	156.29	0.367
IBS	292.24		IBS	140.17	
IBD	**244.56**	**0**.**012**	IBD	**98.67**	**0**.**017**
IBS	**292.24**		IBS	**140.17**	
			IBD I^*^	103.98	0.221
			IBD NI^*^	67.83	

^*^I, inflamed; NI, not Inflamed. *p* values < 0.05 are reported in bold.

β-diversity analyses, performed by unweighted and weighted UniFrac algorithms, performed on all fecal samples, showed the IBD cluster, separated from CTRLs and IBS samples, that resulted intermixed (Supplementary Figures S1A,B). β-diversity analyses, performed on IBD, and CTRLs showed a clear separation between the two groups (PERMANOVA *p* = 0.001 for both analyses) (Supplementary Figures S2, S3A). The same result was obtained forIBD and IBS comparison (PERMANOVA *p* = 0.001 and *p* = 0.002, respectively) (Supplementary Figures S2, S3C). A not significant separation between IBS and CTRLs (PERMANOVA *p* = 0.13 and *p* = 0.053, respectively) was reported (Supplementary Figures S2, S3B).

**FIGURE 1:**
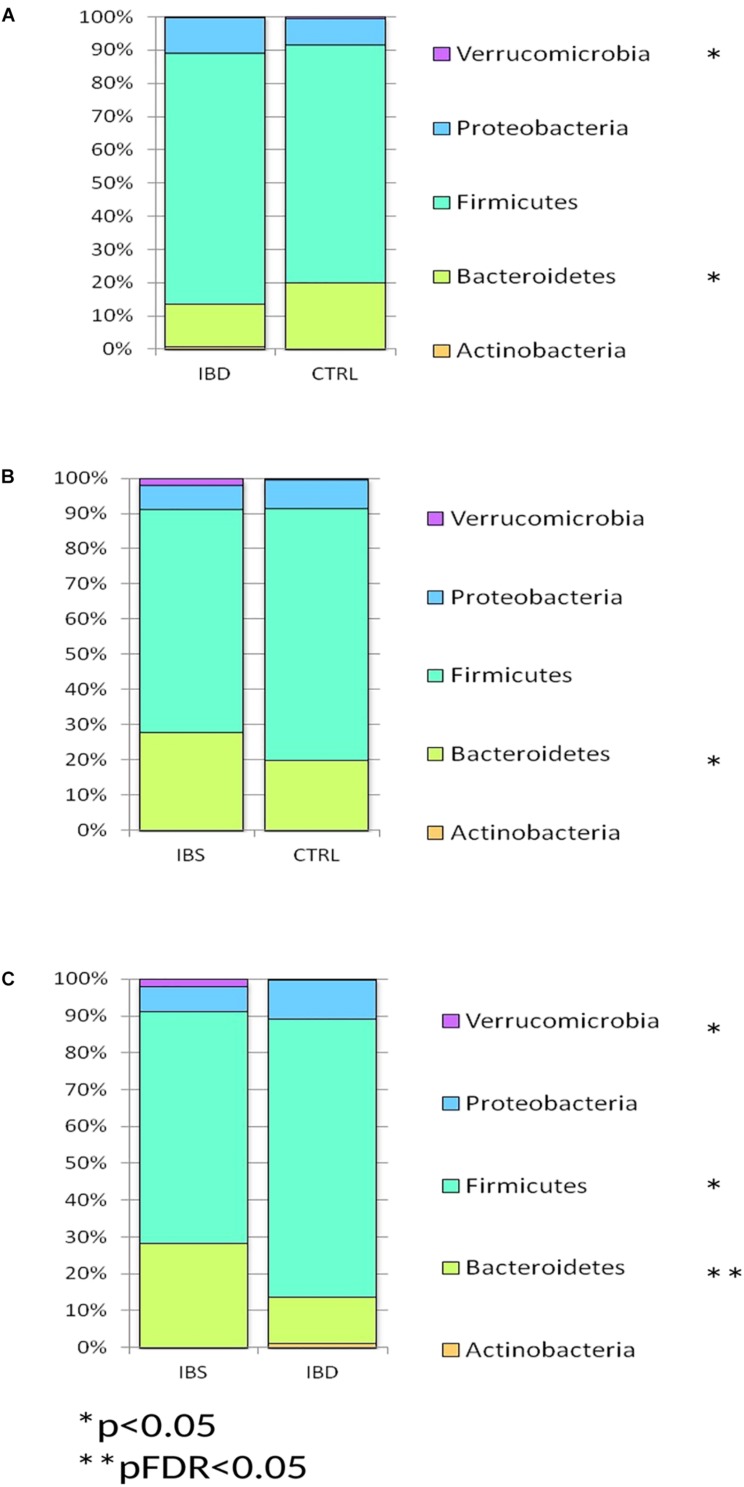
Bar chart reporting Kruskal-Wallis test results on OTUs grouped at taxonomic level of phylum for IBD vs. CTRL **(A)**, IBS vs. CTRL **(B)**, and IBD vs. IBS **(C)**, in fecal samples. Fecal samples have been grouped and averaged in the comparisons IBD vs. CTRL **(A)**, IBS vs. CTRL **(B)**, and IBD vs. IBS **(C)**. Each column in the plot represents a group, and each color in the column represents the percentage of relative abundance for each phyla.

Phylum distribution in IBD harbored less bacteroidetes and Verrucomicrobia (*p* < 0.05) than CTRLs (Figure 1A), while in IBS bacteroidetes appeared increased compared to CTRLs (*p* < 0.05) (Figure 1B). When compared IBS and IBD samples, a significant increase of bacteroidetes (pFDR < 0.05) and Verrucomicrobia (*p* < 0.05) and a reduction of Actinobacteria (*p* < 0.05) was observed in IBS (Figure 1C).

**FIGURE 2:**
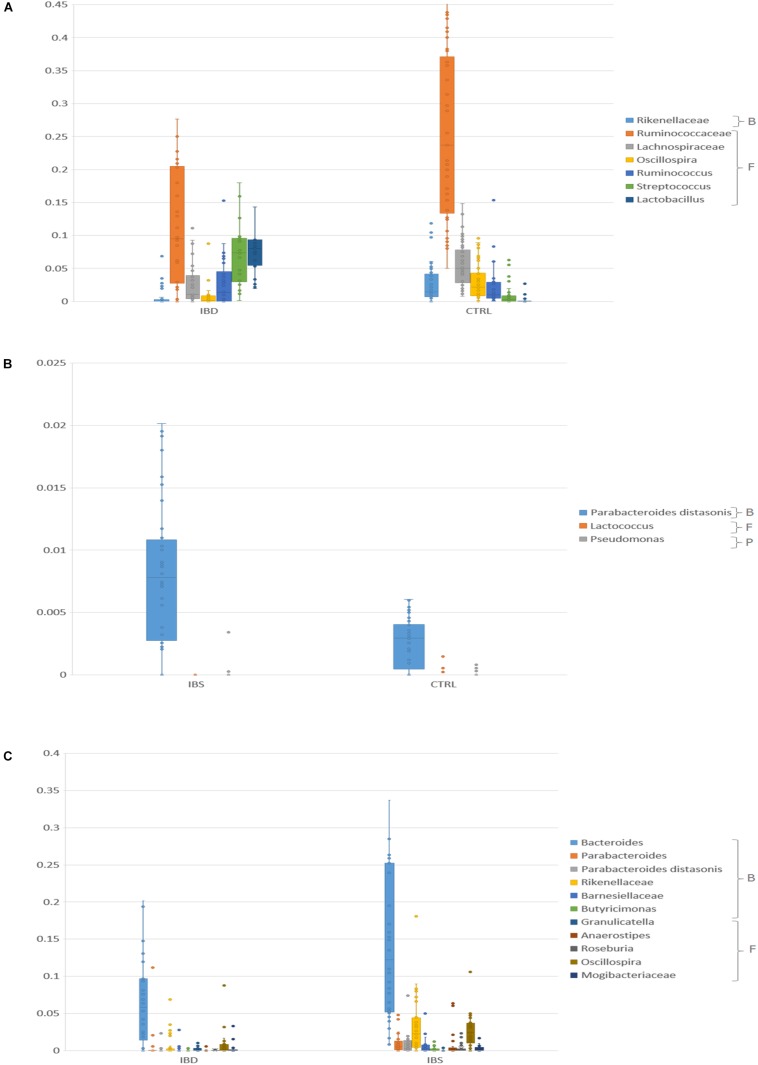
Bar chart reporting Kruskal-Wallis test results on OTUs grouped at taxonomic level of families/species for IBD vs. CTRL **(A)**, IBS vs. CTRL **(B)**, and IBD vs. IBS **(C)**, in fecal samples. Fecal samples have been grouped and averaged in the comparisons. For the IBD vs. CTRL **(A)** comparison, only OTUs that showed pFDR < 0.05 and relative abundance >0.01 were reported. For the IBS vs. CTRL **(B)** only OTUs that showed *p* < 0.05 were reported. For the IBD vs. IBS **(C)** comparison, only OTUs that showed pFDR < 0.05 were reported. B, Bacteroidetes, F, Firmicutes, P, Proteobacteria.

Kruskal-Wallis test showed that *Ruminococcus, Streptococcus, Lactobacillus* were significantly represented in IBD vs. CTRLs (pFDR < 0.05), while Ruminococcaceae, Lachnospiraceae, Rikenellaceae, and *Oscillospira* were underrepresented in IBD (pFDR < 0.05) (Figure 2A and Supplementary Table S1). *Akkermansia muciniphila* was reduced in IBD compared to CTRLs (pFDR < 0.05).

In IBS samples, *Parabacteroides distasonis* was increased, while *Lactococcus* and *Pseudomonas* were reduced compared to CTRLs (*p* < 0.05) (Figure 2B and Supplementary Table S1).

When compared IBS and IBD samples, *Bacteroides*, *Oscillospira*, Rikenellaceae, *Butyricimonas*, *Roseburia*, Mogibacteriaceae, Barnesiellaceae, *Anaerostipes*, and *P. distasonis*, *Parabacteroides* were more abundant in IBS (pFDR < 0.05) than in IBD, while *Granulicatella* was less abundant (Figure 2C and Supplementary Table S1). *A. muciniphila* was reduced in IBD compared to IBS (*p* < 0.05).

### Mucosal Microbiota Composition and Distribution

A total of 130,330.00 sequencing reads were obtained from 142 mucosal samples.

Similarly, to fecal samples, biopsy α-diversity showed decreasing values from CTRLs to IBD through IBS (Table 3). No significant difference was observed between IBD inflamed and not inflamed tissue samples (Table 3).

β-diversity analyses, performed by unweighted and weighted UniFrac algorithms, performed on all mucosal samples, showed a separation between IBD (inflamed tissue) and CTRLs and between IBD (inflamed tissue), and IBS (but a less separation between IBS and CTRLs (Supplementary Figures S4A,B). This result was confirmed by PERMANOVA tests, applied on both Unweighted and Weighted distant matrices, performed separately on IBD (inflamed tissue) vs. CTRLs (*p* = 0.001 for both analyses), IBD vs. IBS (*p* = 0.001 for both analyses), and IBS vs. CTRLs (*p* = 0.092 and *p* = 0.084, respectively) (Supplementary Figures S5, S6A–C). No significant difference was observed between IBD inflamed and not inflamed tissue samples (PERMANOVA *p* value = 0.94 and 0.36, respectively) (Supplementary Figures S5, S6D).

**FIGURE 3:**
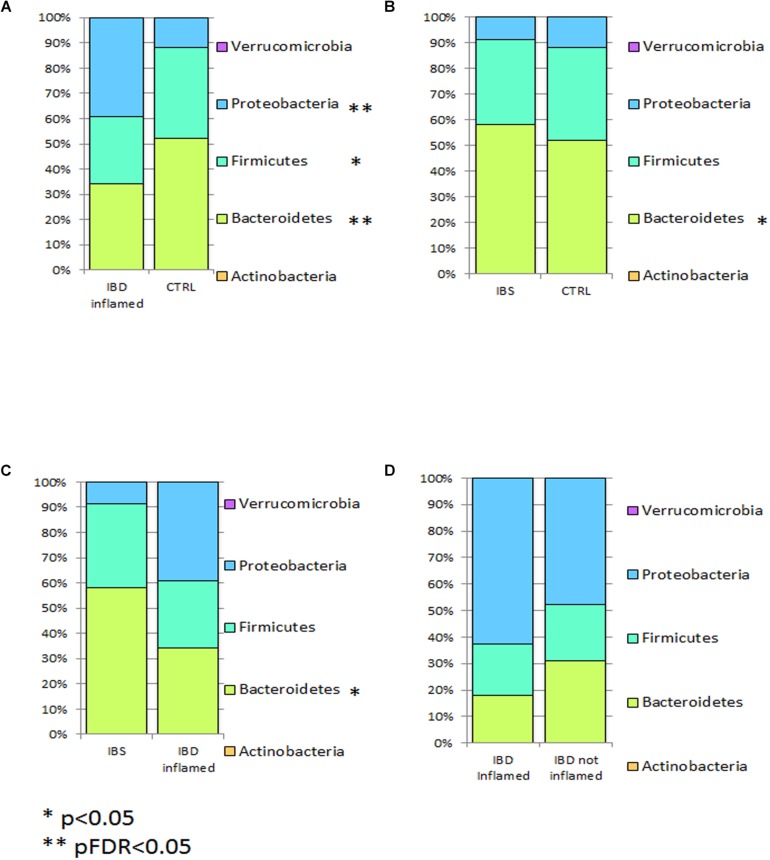
Bar chart reporting Kruskal-Wallis test results on mucosal OTUs grouped at taxonomic level of phylum for IBD vs. CTRL **(A)**, IBS vs. CTRL **(B)**, IBD vs. IBS **(C)**, and IBD inflamed vs. not inflamed **(D)**. Mucosal samples have been grouped and averaged in the comparisons IBD inflamed vs. CTRL **(A)**, IBS vs. CTRL **(B)**, IBD inflamed vs. IBS **(C)**, and IBD inflamed vs. not inflamed **(D)**. Each column in the plot represents a group, and each color in the column represents the percentage of relative abundance for each phyla. *p* values corrected for FDR were highlighted by star.

Inflamed mucosa microbiota of IBD patients harbored more Proteobacteria (pFDR < 0.05), and less bacteroidetes (pFRD < 0.05) and firmicutes (*p* < 0.05) respect to CTRLs (Figure 3A). In IBS, bacteroidetes were increased compared to CTRLs (*p* < 0.05) (Figure 3B) and compared to IBD inflamed mucosa (*p* < 0.05) (Figure 3C). No statistical difference was observed comparing inflamed vs. not inflamed IBD mucosa (Figure 3D).

**FIGURE 4:**
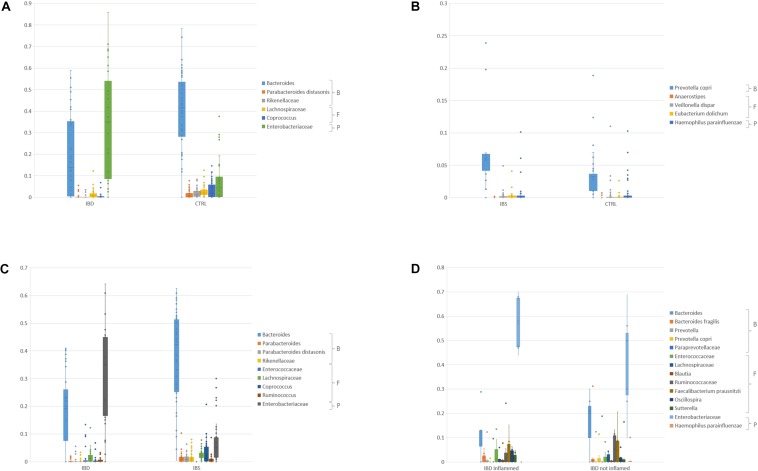
Bar chart reporting Kruskal-Wallis test results on mucosal OTUs of families/species for IBD inflamed vs. CTRL **(A)**, IBS vs. CTRL **(B)**, IBD inflamed vs. IBS **(C),** and IBD inflamed vs. not inflamed **(D)**. For the IBD inflamed vs. CTRL **(A)** comparison only OTUs that showed pFDR < 0.05 and relative abundance >0.01 were reported. For the IBS vs. CTRL **(B)** only OTUs that showed *p* < 0.05 were reported. For the IBD vs. IBS **(C)** comparison, only OTUs that showed pFDR < 0.05 and relative abundance >0.01 were reported. For the IBD inflamed vs. not inflamed biopsies only OTUs that showed a relative abundance >0.01 were reported **(D)**. B, Bacteroidetes, F, Firmicutes, P, Proteobacteria.

At genus/species level, an increase of Enterobacteriaceae (pFDR < 0.05) and a reduction of *Bacteroides*, *P. distasonis*, Rikenellaceae, *Coprococcus*, and Lachnospiraceae were observed in IBD inflamed mucosa compared to CTRLs (pFDR < 0.05) (Figure 4A and Supplementary Table S2). Moreover, also *Faecalibacterium prausnitzii* and Ruminococcaceae were decreased in IBD (*p* < 0.05). An increment of *Prevotella copri*, *Eubacterium dolichum*, *Veillonella dispar*, and *Haemophilus parainfluenzae*, and a reduction of *Anaerostipes* were reported in IBS samples compared to CTRLs (*p* < 0.05) (Figure 4B and Supplementary Table S2). In the comparison between IBS and IBD inflamed mucosa, *Bacteroides*, Lachnospiraceae, *Parabacteroides*, *P. distasonis*, Rikenellaceae, *Coprococcus*, and *Ruminococcus* appeared increased in IBS, though Enterobacteriaceae, Enterococcaceae were reduced in IBS respect to IBD (Figure 4C and Supplementary Table S2). The intra-individual comparison between inflamed vs. not inflamed IBD mucosa was focused only on the OTUs with relative abundance >0.01. In this comparison *Bacteroides*, Ruminococcaceae, *Bacteroides fragilis*, *Sutterella*, Paraprevotellaceae, *Faecalibacterium prausnitzii*, *H. parainfluenzae*, Lachnospiraceae, and *P. copri* were decreased in IBD inflamed mucosa. Enterobacteriaceae, *Prevotella*, Enterococcaceae, *Oscillospira*, and *Blautia* were increased in IBD, even if the *p* value was not significant (Figure 4D and Supplementary Table S2).

### Mucosa Versus Stool Microbiota Comparison

Unweighted and Weighted β-diversity analyses showed a clear and significant separation between mucosa and stool samples in all groups (Supplementary Figures S7, S8A–C). At phylum level, firmicutes and Actinobacteria distribution were increased, while bacteroidetes and Proteobacteria resulted decreased in stools from all groups (pFDR < 0.05, data not shown). Verrucomicrobia resulted higher in stool compared to mucosal samples (pFDR < 0.05 for CTRL and IBS). When considering only stool samples Verrucomicrobia was more abundant in IBS samples.

Microbiota composition (filtered for pFDR < 0.05 and relative abundance >0.01), showed similar profile in CTRLs, and IBS for both mucosal and stool samples. IBD pattern was characterized by a different and specific signature.

In particular, in CTRL and IBS, *Bacteroides* and Lachnospiraceae were higher in mucosal samples, while Clostridiaceae, *Oscillospira*, Ruminococcaceae, and *Sutterella* were higher in stools (Supplementary Figure S9 and Supplementary Table S3). In IBD, *Blautia*, Clostridiaceae, *Coprococcus*, Erysipelotrichaceae, *Lactobacillus*, and *Streptococcus* were higher in stools respect to mucosal samples (*p* < 0.05) (Supplementary Figure S9 and Supplementary Table S3).

### Comparison of Taxonomic Composition According to Disease Status by LEfSe for 16S rRNA-Based Metagenomic Biomarker

The linear discriminant analysis effect size analysis on the taxonomic composition performed on biopsy samples and comparing all groups showed a higher number of OTUs featuring the IBD/CTRL pair (Supplementary Figures S10A–C).

**FIGURE 5:**
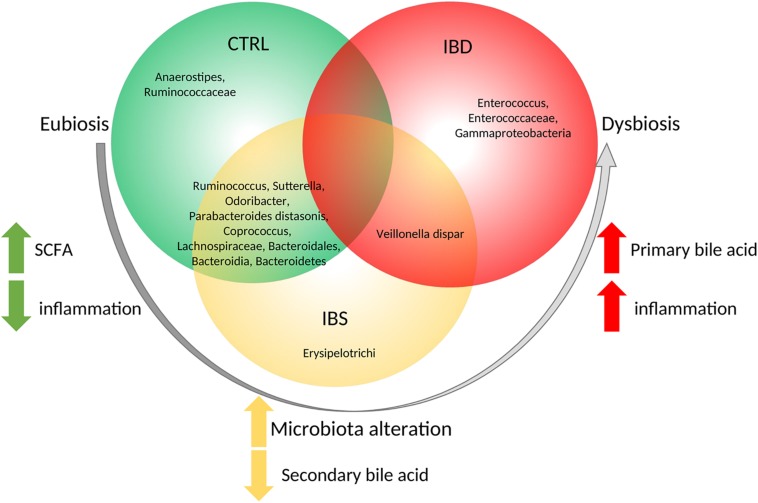
Descriptive model of microbiota composition and its role starting from eubiosis to dysbiosis based on biopsy samples. Model suggests that, in eubiosis condition, specific OTUs compose microbiota and maintain equilibrium. A microbiota alteration trigs the inflammation, leading to an increment of Erysipelotrichi and a reduction of secondary bile acid production. Then a further increment of inflammation leads to increase of primary bile producers with the consequence of dysbiosis.

Considering LEfSe results, we selected the OTUs exclusively associated to each group, or shared between different groups (Figure 5). The *Anaerostipes* and Ruminococcaceae were identified as potential biomarkers for CTRL microbiota Erysipelotrichi for IBS and Gammaproteobacteria, *Enterococcus*, Enterococcaceae for IBD (Figure 5). *V. dispar* was identified both in IBS and IBD, meanwhile *Ruminococcus*, *Sutterella*, *Odoribacter*, *P. distasonis*, *Coprococcus*, Lachnospiraceae, Bacteroidales, Bacteroidia, and Bacteroidetes were identified in CTRLs as well as in IBS. LEfSe analysis confirmed the results of Kruskal-Wallis test for Ruminococcaceae, *P. distasonis*, *Coprococcus* and Lachnospiraceae.

### Gut Microbial Profiling and Clinical Features in IBD and IBS

Unweighted and Weighted β-diversity analyses of IBD biopsy and fecal samples does not revealed a clustering between CD and UC samples (PERMANOVA test on Unweighted analysis *p* = 0.176 and *p* = 0.109, respectively; PERMANOVA test on Weighted analysis *p* = 0.226 and *p* = 0.096, respectively) (Supplementary Figures S11, S12A,B). Moreover, stratifying IBD samples for disease activities we do not obtain any statistical clustering (PERMANOVA test on Unweighted analysis *p* = 0.66 and *p* = 0.12, respectively: PERMANOVA test on Weighted analysis *p* = 0.176 and *p* = 0.686, respectively) (Supplementary Figures S11, S12C,D). Appling β-diversity analysis on IBS biopsy and fecal samples stratified for predominant bowel habits, we not obtain a clustering amongst diarrhea, constipation, and alternating sample group (PERMANOVA test on Unweighted analysis *p* = 0.206 and *p* = 0.091, respectively; PERMANOVA test on Unweighted analysis *p* = 0.509 and *p* = 0.251) (Supplementary Figures S13, S14A,B).

## Discussion

In this study fecal and mucosal microbiota were characterized by 16S rRNA gene amplicons, in a large cohort of IBS and IBD patients compared to CTRLs. These subjects were enrolled from 2015 to 2017 in two hospitals in Rome.

The relatively low number of patients enrolled, considering the heterogeneity of the IBD and IBS populations and the presence of possibly confounding factors such as medications and diet represents a limitation of this study.

Several data available in literature reported different microbiota profiles in IBD and IBS patients, but those comparing mucosal and fecal microbiota are still lacking or controversial. This study could contribute to fill the gap of knowledge about the role of mucosal and fecal microbiota in inflammation in IBS or IBD patients.

As observed in previous studies (Ponnusamy et al., 2011; Shaw et al., 2016; Botschuijver et al., 2017; Hirano et al., 2018), the microbiota ecological analyses revealed a significant decrease in richness related to the increasing bowel inflammation (CTRLs > IBS > IBD). Moreover, the β-diversity analyses and the phylum profiling revealed a more different microbiota profile between IBD and CTRL, respect to IBS and CTRL. These results could reflect the increasing inflammatory bowel status observed going from CTRL toward IBS and to IBD.

Moreover, our results showed that the mucosa associate bacteria are equally distributed between inflamed and not-inflamed tissue in the intra-individual comparison. Bibiloni et al., 2006, analyzing inflamed and non-inflamed biopsies by denaturing gradient gel electrophoresis (DGGE), reported high similar bacterial profiles between the two sample groups (Bibiloni et al., 2006). The same conclusions were reported by other authors that analyzed biopsy samples of ulcerated and non-ulcerated mucosa of IBD patients by temporal temperature gradient gel electrophoresis (TTGE) (Seksik et al., 2005; Sokol et al., 2007; Vasquez et al., 2007). Finally, also Gophna et al., 2017, by high-throughput sequencing of 16S rRNA cloned libraries, reported that there is not a localized dysbiosis in IBD between inflamed and non-inflamed tissue (Gophna et al., 2017). Indeed, in line with our study, the differences in bacterial composition were not due to the inflamed condition of the tissue but the bacteria associated with the mucosal surface seems to be related to the specific disease and to the systemic inflammation condition.

In controls, the most represented species of fecal microbiota were Ruminococcaceae, *Oscillospira* and Lachnospiraceae as supported by other studies (Maukonen et al., 2015; Santoru et al., 2017; Altomare et al., 2018).

In patients with IBS the fecal microbiota was characterized by the presence of *Oscillospira*, which, interestingly, has been already described in normal mucosa or in case of mild inflammation (Gophna et al., 2017).

We showed the reduction of *A. muciniphila* in fecal microbiota of IBD patients, compared to CTRLs and IBS, as also previously reported (Bajer et al., 2017). This species exerts beneficial effects in the host (Everard et al., 2013; Derrien et al., 2017; Ottman et al., 2017). It has been found negatively correlated with IBD (Png et al., 2010; Rajilić-Stojanović et al., 2013) and IBS (Gobert et al., 2016), suggesting its protective role when abundant in the microbiota composition. Furthermore, in an animal model harboring a human intestinal microbiota, the presence of this microorganism reduces colonic histological damages, and tissue mRNA expression of pro-inflammatory mediators (Gobert et al., 2016).

When looking at mucosal microbial composition, *V. dispar*, *P. copri* and *H. parainfluenzae* were significantly represented only in IBS mucosal microbiota. Regarding *V. dispar*, it has generally been considered a non-pathogenic bacteria, but recently, Kasai and co-workers suggested a possible role in inflammation and in colorectal cancer (Kasai et al., 2016). Moreover, *P. copri* has been associated to enhancing susceptibility to inflammatory disorders like arthritis through intrinsic Th17 promoting capability, driving cytokines IL-6 and IL-23 (Scher et al., 2013) and has been associated to systemic inflammation status too (Creely et al., 2007; Pedersen et al., 2016). A recent study showed that *P. copri* enhances dextran sulfate sodium-induced colitis in mice, in association with increased IFN-γ production, suggesting that *P. copri* promotes Th1 immune responses in experimental colitis (Larsen, 2017).

In our study Enterobacteriaceae and *Streptococcus* were associated to IBD microbiota. The possible involvement of *Streptococcus* in the inflammatory status of IBD was already suggested by other studies that reported the interaction of streptococcal virulence factors with immune cells eliciting inflammatory response in different organs (Herrera et al., 2009; Rooks et al., 2014).

Regarding Enterobacteriaceae, previous studies have found elevated abundance of this family in Crohn’s Disease patients (Swidsinski et al., 2005; Gophna et al., 2006; Baumgart et al., 2007; Sepehri et al., 2011; Jensen et al., 2015; Palmela et al., 2018), supporting our data.

By the LEfSe analyses we propose a model based on the potential bacterial biomarkers associated to mucosal inflammation. In this model (Figure 5), *Anaerostipes* and Ruminococcaceae were exclusively associated to CTRL microbiota. The healthy role of these bacteria is probably exerted through the production of short chain fatty acids (SCFA) (Lozupone et al., 2012; den Besten et al., 2013). Interestingly, CTRL and IBS share the presence of *Ruminococcus*, *Sutterella*, *Odoribacter*, *P. distasonis*, *Coprococcus*, Lachnospiraceae, and Bacteroidales that are commonly recognized as commensals (Unno et al., 2015; Hiippala et al., 2016). Moreover, Wang and co-workers correlated high levels of *Odoribacter*, *Sutterella*, *Coprococcus*, *Lachnospira*, and *Ruminococcus* with the improvement of health status in CD patients, leading to suppose a positive role against gut inflammation (Wang et al., 2018). Erysipelotrichi were exclusively present in IBS mucosal samples. Of interest, overgrowth of several bacteria such as Erysipelotrichi is induced, in an animal model, by administration of cholic acid, which has been reported to be increased in IBS patients (Islam et al., 2011; Duboc et al., 2012).

The mucosal microbiota of IBS shares the presence of *V. dispar* with IBD. *V. dispar* showed the ability to degrade cholate and deoxycholate in secondary products (Aries et al., 1969; Duboc et al., 2012). The association of this species to IBD and IBS suggests a role of these bacteria in dysmetabolism of bile acids reported in IBD and IBS (Dior et al., 2016). Actually, in IBD patients was reported a decrease of secondary biliary acids with a related loss of their anti-inflammatory effects, thus suggesting biliary acids as important players in the pro-inflammatory processes (Duboc et al., 2013).

Finally, we reported that *Enterococcus*, Enterococcaceae, Gammaproteobacteria are exclusively present in IBD patients. It is well known that *Enterococcus faecalis* was the only human commensal to induce IBD (Nell et al., 2010); and that the Gammaproteobacteria (Frank et al., 2011) (e.g., *E. coli* AIEC, *Klebsiella* spp., *Pseudomonas* spp., and *Salmonella*) overgrew in mucosa of IBD patients (Nagao-Kitamoto and Kamada, 2017).

The knowledge of microbiota composition in patients with IBD and IBS is largely debated. In this panorama, this study provides an overview of the alterations of microbiota in stool and mucosal samples from IBD and IBS patients in relation of inflammation grading existing between the two diseases. Moreover, the potential mucosal biomarkers identified in this study, could be evaluated as actors in the IBD and IBS intestinal inflammation and then considered in the development of new clinical interventions for the prevention and treatment of IBD and IBS, based on microbiota modulation.

## Ethics Statement

This study was carried out in accordance with the recommendations of the study protocol “Tor Vergata” General Hospital GR-2011-02350817 Register of Experiments 44/15, University of Rome Tor Vergata; Campus Prot. 24/15 PAR ComEt CBM, Campus Bio-Medico University with written informed consent from all subjects. All subjects gave written informed consent in accordance with the Declaration of Helsinki. The protocol was approved by the University of Rome Tor Vergata and Campus Bio-Medico University committees.

## Author Contributions

ALP, MG, MiC, MaC, SA, FDC, AnA, FZ, GM, and LB designed the study. FDC, ALP, AnA, FZ, AR, SR, and FDB analyzed the data. AnA, FZ, SC, EC, AlA, and EC collected the samples. ALP, MG, and FZ acquired the funding. ALP, FDC, FZ, MG, and AnA wrote the original draft of the manuscript. MaC, MG, MiC, LP, and GM supervised writing of the manuscript. ALP, FDC, FZ, AnA, FDB, AR, EC, SR, EC, LB, GM, MiC, SA, MaC, LP, and MG contributed by reviewing and editing the manuscript. All authors approved the final version of the manuscript as submitted and agreed to be accountable for all aspects of the work.

## Conflict of Interest Statement

The authors declare that the research was conducted in the absence of any commercial or financial relationships that could be construed as a potential conflict of interest.

## Footnotes

1 http://qiime.org/scripts/identify_chimeric_seqs.html

2 https://www.ncbi.nlm.nih.gov/bioproject/?term=PRJNA391149
