# Supplementary material for: Fecal and Mucosal Microbiota Profiling in Irritable Bowel Syndrome and Inflammatory Bowel Disease
Source: Front Microbiol. 2019 Jul 17;10:1655. doi: 10.3389/fmicb.2019.01655 (PMC6650632; doi:10.3389/fmicb.2019.01655)
Supplement: Supplementary file 1 [file Data_Sheet_1.pdf]

Supporting Material

Fecal and Mucosal microbiota profiling in Irritable bowel syndrome and Inflammatory Bowel Disease

Alessandra Lo Presti[1#]*, Francesca Zorzi[2#], Federica Del Chierico[3]*, Annamaria Altomare[4], Silvia Cocca[4], Alessandra Avola[4], Fabiola De Biasio[4], Alessandra Russo[3], Eleonora Cella[5], Sofia Reddel[3], Emma Calabrese[2], Livia Biancone[2], Giovanni Monteleone[2], Michele Cicala[4], Silvia Angeletti[6], Massimo Ciccozzi[5], Lorenza Putignani[7°], Michele Pier Luca Guarino[4°]

[1] Department of Infectious Diseases, Istituto Superiore di Sanità, Rome, Italy
[2] Gastrointestinal Unit, Department of Systems Medicine, University Tor Vergata, Rome, Italy
[3] Human Microbiome Unit, Bambino Gesù Children's Hospital, IRCCS, Rome, Italy
[4] Unit of Digestive Disease, Campus Bio Medico University, Rome, Italy
[5] Unit of Medical Statistics and Molecular Epidemiology, University Campus Bio-Medico, Rome, Italy
[6] Unit of Clinical Laboratory Science, University Campus Bio-Medico, Rome, Italy
[7]Human microbiome Unit and Parasitology Unit, Bambino Gesù Children's Hospital, IRCCS, Rome, Italy

# These authors contributed equally
° These authors contributed equally (co-senior authors)

S1 Table. Kruskal-Wallis test on faecal samples. In table are reported the average values of each OTUs for each group. For the IBD versus CTRL comparison only OTUs that showed pFDR<0.05 and relative abundance >0.01 were reported. For the IBS versus CTRL only OTUs that showed p<0.05 were reported. For the IBD versus IBS comparison only OTUs that showed pFDR<0.05 were reported.

**Phylum**	**Family**	**IBD**	**CTRL**	**P value**	**pFDR**
Bacteroidetes	Rikenellaceae	0.00638	0.02683	0.00000	0.00013
Firmicutes	Ruminococcaceae	0.11048	0.25211	0.00006	0.00288
Firmicutes	Lachnospiraceae	0.02614	0.05423	0.00267	0.03886
**Phylum**	**Genus/Species**	**IBD**	**CTRL**	**P value**	**pFDR**
Firmicutes	*Oscillospira*	0.00784	0.03036	0.00000	0.00013
Firmicutes	*Ruminococcus*	0.02808	0.02568	0.00371	0.04504
Firmicutes	*Streptococcus*	0.07250	0.00836	0.00098	0.01931
Firmicutes	*Lactobacillus*	0.07215	0.00148	0.00130	0.02229
**Phylum**	**Genus/Species**	**IBS**	**CTRL**	**P value**	**pFDR**
Firmicutes	*Lactococcus*	0.00000	0.00008	0.03146	0.92257
Bacteroidetes	*Parabacteroides distasonis*	0.00801	0.00279	0.03343	0.92257
Proteobacteria	*Pseudomonas*	0.00010	0.00011	0.04235	0.92257
**Phylum**	**Family**	**IBS**	**IBD**	**P value**	**pFDR**
Bacteroidetes	Rikenellaceae	0.03195	0.00638	0.00000	0.00028
Firmicutes	Mogibacteriaceae	0.00373	0.00213	0.00044	0.01409
Bacteroidetes	Barnesiellaceae	0.00569	0.00149	0.00017	0.00611
**Phylum**	**Genus/Species**	**IBS**	**IBD**	**P value**	**pFDR**
Bacteroidetes	*Bacteroides*	0.14358	0.06649	0.00160	0.04240
Bacteroidetes	*Butyricimonas*	0.00203	0.00012	0.00003	0.00154
Bacteroidetes	*Parabacteroides distasonis*	0.00801	0.00106	0.00000	0.00028
Bacteroidetes	*Parabacteroides*	0.00954	0.00507	0.00002	0.00135
Firmicutes	*Oscillospira*	0.02601	0.00784	0.00001	0.00085
Firmicutes	*Granulicatella*	0.00022	0.00158	0.00064	0.01875
Firmicutes	*Roseburia*	0.00281	0.00039	0.00200	0.04844
Firmicutes	*Anaerostipes*	0.00591	0.00048	0.00015	0.00611

S2 Table. Kruskal-Wallis test on biopsy samples. In table are reported the average values of each OTUs for each group. For the IBD inflamed versus CTRL comparison only OTUs that showed pFDR<0.05 and relative abundance >0.01 were reported. For the IBS versus CTRL only OTUs that showed p<0.05 were reported. For the IBD versus IBS comparison, only OTUs that showed pFDR<0.05 and relative abundance >0.01 were reported. For the IBD inflamed versus IBD not inflamed biopsies only OTUs that showed a relative abundance >0.01 were reported.

Phylum	Family	IBD	CTRL	P value	pFDR
Bacteroidetes	Rikenellaceae	0.00366	0.01924	0.00000	0.00009
Firmicutes	Lachnospiraceae	0.03005	0.05629	0.00001	0.00037
Proteobacteria	Enterobacteriaceae	0.34403	0.08332	0.00007	0.00265
**Phylum**	**Genus/Species**	**IBD**	**CTRL**	**P value**	**pFDR**
Bacteroidetes	*Bacteroides*	0.19019	0.41837	0.00000	0.00009
Bacteroidetes	*Parabacteroides distasonis*	0.00492	0.01412	0.00009	0.00298
Firmicutes	*Coprococcus*	0.00461	0.03435	0.00000	0.00008
**Phylum**	**Genus/Species**	**IBS**	**CTRL**	**P value**	**pFDR**
Bacteroidetes	*Prevotella copri*	0.06808	0.00439	0.03209	0.86937
Firmicutes	*Anaerostipes*	0.00033	0.00075	0.01004	0.73069
Firmicutes	*Eubacterium dolichum*	0.00334	0.00054	0.00323	0.73069
Firmicutes	*Veillonella dispar*	0.00572	0.00049	0.03477	0.86937
Proteobacteria	*Haemophilus parainfluenzae*	0.00719	0.00082	0.02662	0.86937
**Phylum**	**Family**	**IBS**	**IBD**	**P value**	**pFDR**
Bacteroidetes	Rikenellaceae	0.01479	0.00366	0.00006	0.00303
Firmicutes	Lachnospiraceae	0.06494	0.03005	0.00173	0.03347
Firmicutes	Enterococcaceae	0.00000	0.01714	0.00012	0.00485
Proteobacteria	Enterobacteriaceae	0.05936	0.34403	0.00005	0.00297
**Phylum**	**Genus/Species**	**IBS**	**IBD**	**P value**	**pFDR**
Bacteroidetes	*Bacteroides*	0.37080	0.19019	0.00002	0.00216
Bacteroidetes	*Parabacteroides*	0.01214	0.00320	0.00052	0.01691
Bacteroidetes	*Parabacteroides distasonis*	0.01098	0.00492	0.00005	0.00297
Firmicutes	*Coprococcus*	0.02978	0.00461	0.00000	0.00040
Firmicutes	*Ruminococcus*	0.01064	0.00435	0.00071	0.02078
**Phylum**	**Family**	**IBD Inflamed**	**IBD not inflamed**	**P value**	**pFDR**
Firmicutes	Ruminococcaceae	0.03628	0.04559	0.74640	1.00000
Bacteroidetes	Paraprevotellaceae	0.01070	0.02094	0.93557	1.00000
Firmicutes	Lachnospiraceae	0.00369	0.02803	0.38006	1.00000
Proteobacteria	Enterobacteriaceae	0.58470	0.43399	0.40130	1.00000
Firmicutes	Enterococcaceae	0.02638	0.01372	0.79259	1.00000
**Phylum**	**Genus/Species**	**IBD Inflamed**	**IBD not inflamed**	**P value**	**pFDR**
Bacteroidetes	*Bacteroides*	0.12581	0.19253	0.49507	1.00000
Bacteroidetes	*Bacteroides fragilis*	0.01205	0.03758	0.88025	1.00000
Bacteroidetes	*Prevotella copri*	0.00169	0.01568	0.80841	1.00000
Bacteroidetes	*Prevotella*	0.01522	0.01385	0.63428	1.00000
Firmicutes	*Faecalibacterium prausnitzii*	0.03435	0.04715	0.96238	1.00000
Firmicutes	*Oscillospira*	0.01796	0.01319	0.89146	1.00000

Firmicutes	*Blautia*	0.01052	0.00165	0.30874	1.00000
Proteobacteria	*Haemophilus parainfluenzae*	0.00000	0.01192	0.14561	1.00000
Proteobacteria	*Sutterella*	0.01163	0.02130	0.88025	1.00000

S3 Table. Kruskal-Wallis test on biopsy versus stool samples in CTRL, IBS and IBD groups, respectively. In table are reported the average values of each OTUs for each group, filtered for pFDR<0.05 and relative abundance > 0.01.

**CTRL**					
**Phylum**	**OTUs**	**Biopsy**	**Stool**	**P value**	**pFDR**
Bacteroidetes	*Bacteroides*	0.41837	0.10835	0.00000	0.00000
Bacteroidetes	*Bacteroides fragilis*	0.01632	0.01514	0.00165	0.01851
Bacteroidetes	*Parabacteroides distasonis*	0.01412	0.00279	0.00462	0.04206
Firmicutes	Clostridiaceae	0.00050	0.01060	0.00000	0.00000
Firmicutes	Lachnospiraceae	0.02992	0.01368	0.00018	0.00345
Firmicutes	*Oscillospira*	0.01173	0.03036	0.00072	0.00951
Firmicutes	Ruminococcaceae	0.05679	0.25211	0.00000	0.00000
Firmicutes	*Ruminococcus*	0.00885	0.02568	0.00135	0.01567
Proteobacteria	*Sutterella*	0.02039	0.00326	0.00000	0.00001
**IBS**					
**Phylum**	**OTUs**	**Biopsy**	**Stool**	**P value**	**pFDR**
Bacteroidetes	*Bacteroides*	0.37080	0.14358	0.00000	0.00002
Bacteroidetes	Rikenellaceae	0.01479	0.03195	0.00492	0.04716
Firmicutes	*Blautia*	0.00916	0.01752	0.00169	0.02137
Firmicutes	Clostridiaceae	0.00035	0.01074	0.00000	0.00000
Firmicutes	Lachnospiraceae	0.03949	0.01074	0.00005	0.00111
Firmicutes	*Oscillospira*	0.01279	0.02601	0.00234	0.02660
Firmicutes	Ruminococcaceae	0.04894	0.20046	0.00000	0.00000
Firmicutes	*Streptococcus*	0.00667	0.01082	0.00014	0.00257
Proteobacteria	Sutterella	0.01572	0.00682	0.00000	0.00004
Verrucomicrobia	*Akkermansia muciniphila*	0.00008	0.01884	0.00016	0.00269
**IBD**					
**Phylum**	**OTUs**	**Biopsy**	**Stool**	**P value**	**pFDR**
Firmicutes	*Blautia*	0.00730	0.02622	0.00055	0.01062
Firmicutes	Clostridiaceae	0.00074	0.02390	0.00007	0.00248
Firmicutes	*Coprococcus*	0.00461	0.01234	0.00020	0.00527
Firmicutes	Erysipelotrichaceae	0.00173	0.03138	0.00188	0.02882
Firmicutes	*Lactobacillus*	0.00080	0.07215	0.00000	0.00022
Firmicutes	*Streptococcus*	0.00103	0.07250	0.00000	0.00000

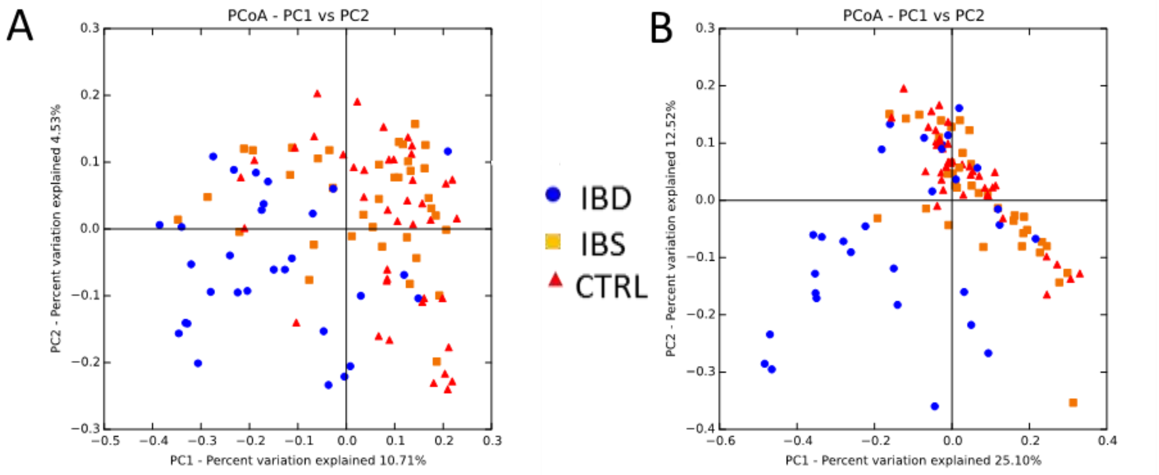

Figure S1. PCoA plot of faecal (A and B) samples stratified in IBD, IBS and CTRL. The plots show the first two principal coordinates (axes) for PCoA using unweighted UniFrac algorithm (A) and weighted UniFrac algorithm (B).

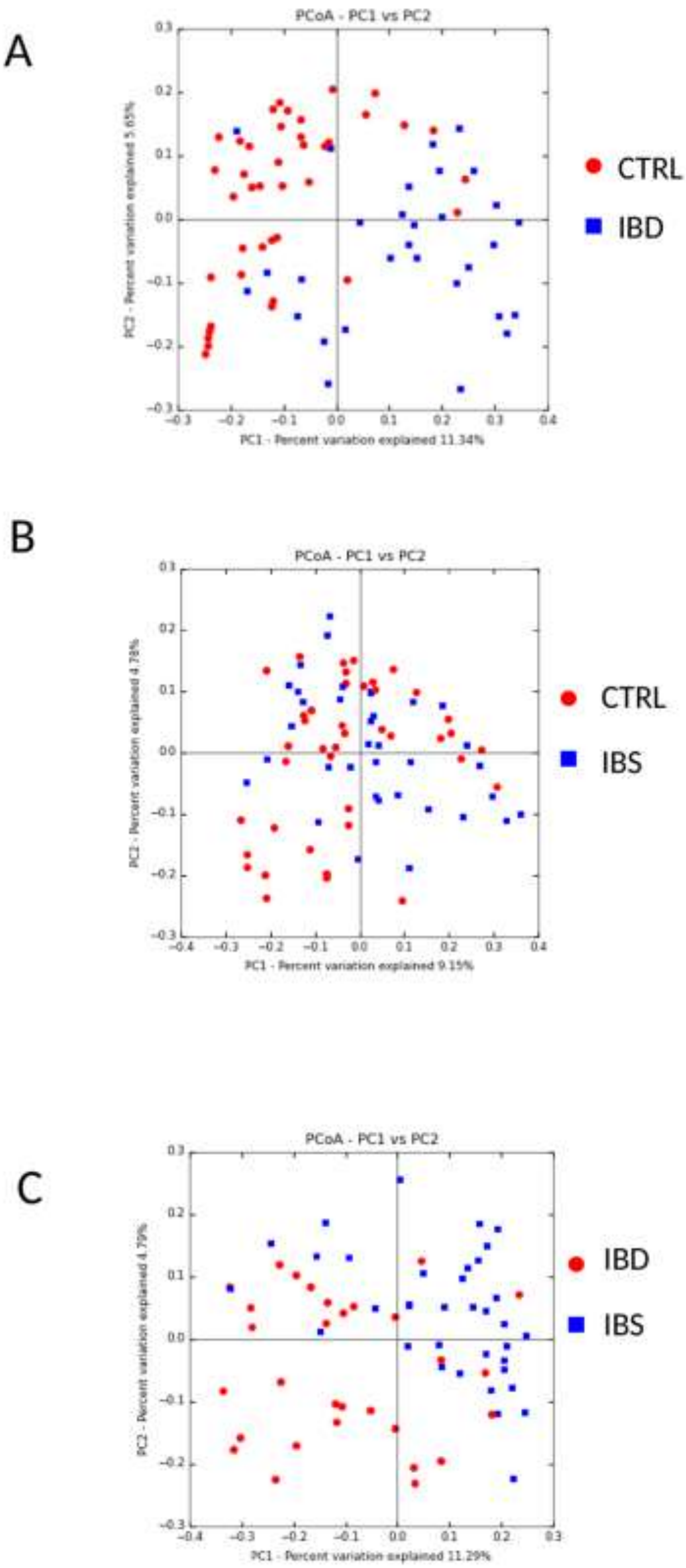

Figure S2. PCoA plot of fecal samples of IBD and CTRL groups (A), IBS and CTRL (B) and IBD and IBS (C). The plots show the first two principal coordinates (axes) for PCoA using unweighted UniFrac algorithm.

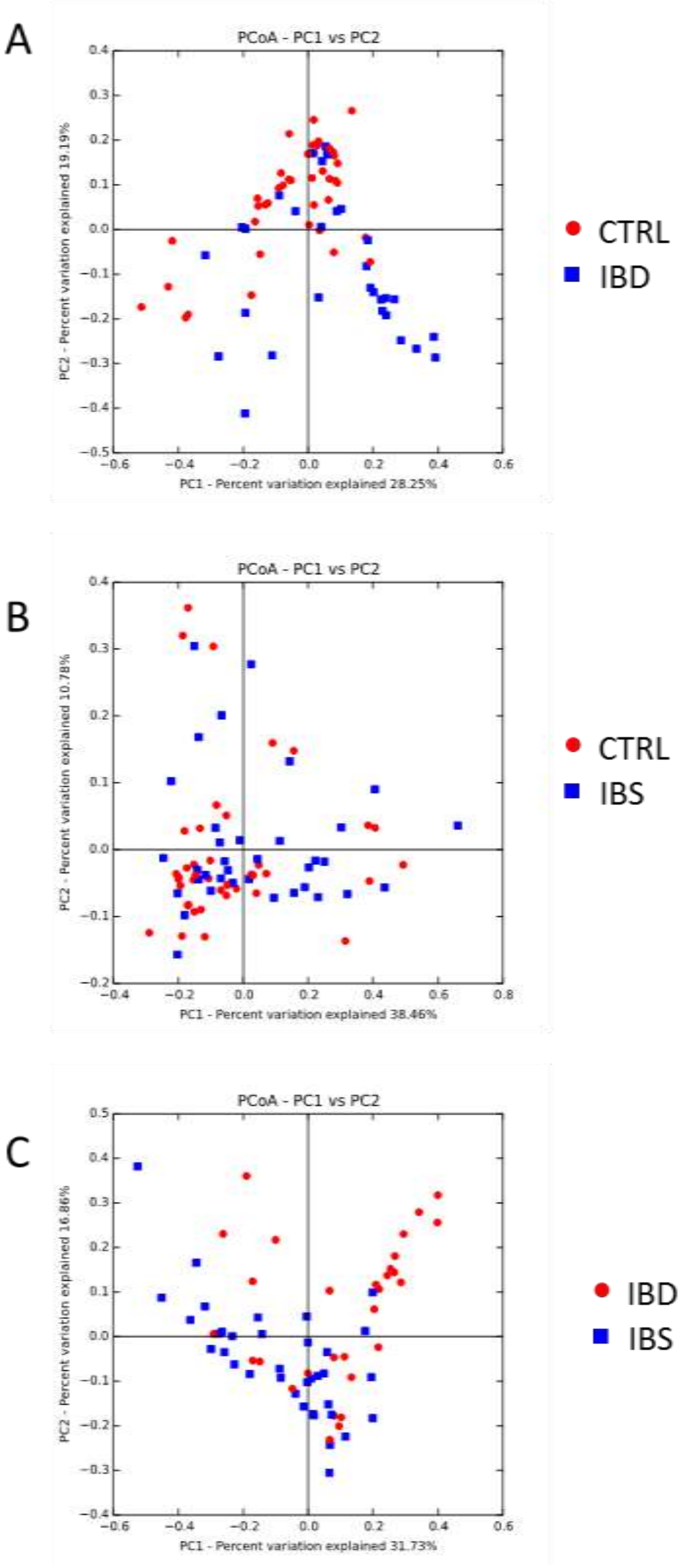

Figure S3. PCoA plot of fecal samples of IBD and CTRL groups (A), IBS and CTRL (B) and IBD and IBS (C). The plots show the first two principal coordinates (axes) for PCoA using weighted UniFrac algorithm.

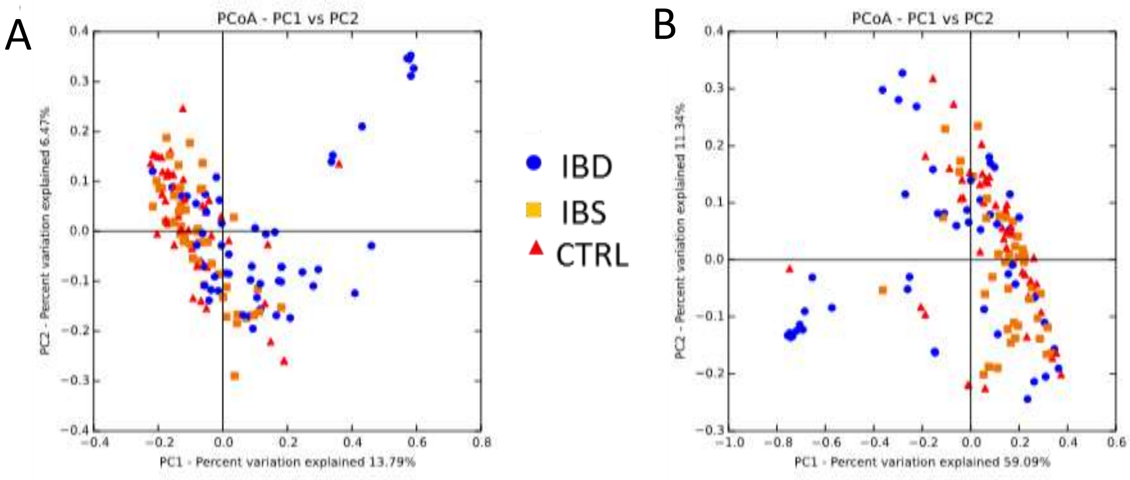

Figure S4. PCoA plot of mucosal (A and B) samples stratified in IBD, IBS and CTRL. The plots show the first two principal coordinates (axes) for PCoA using unweighted UniFrac algorithm (A) and weighted UniFrac algorithm (B).

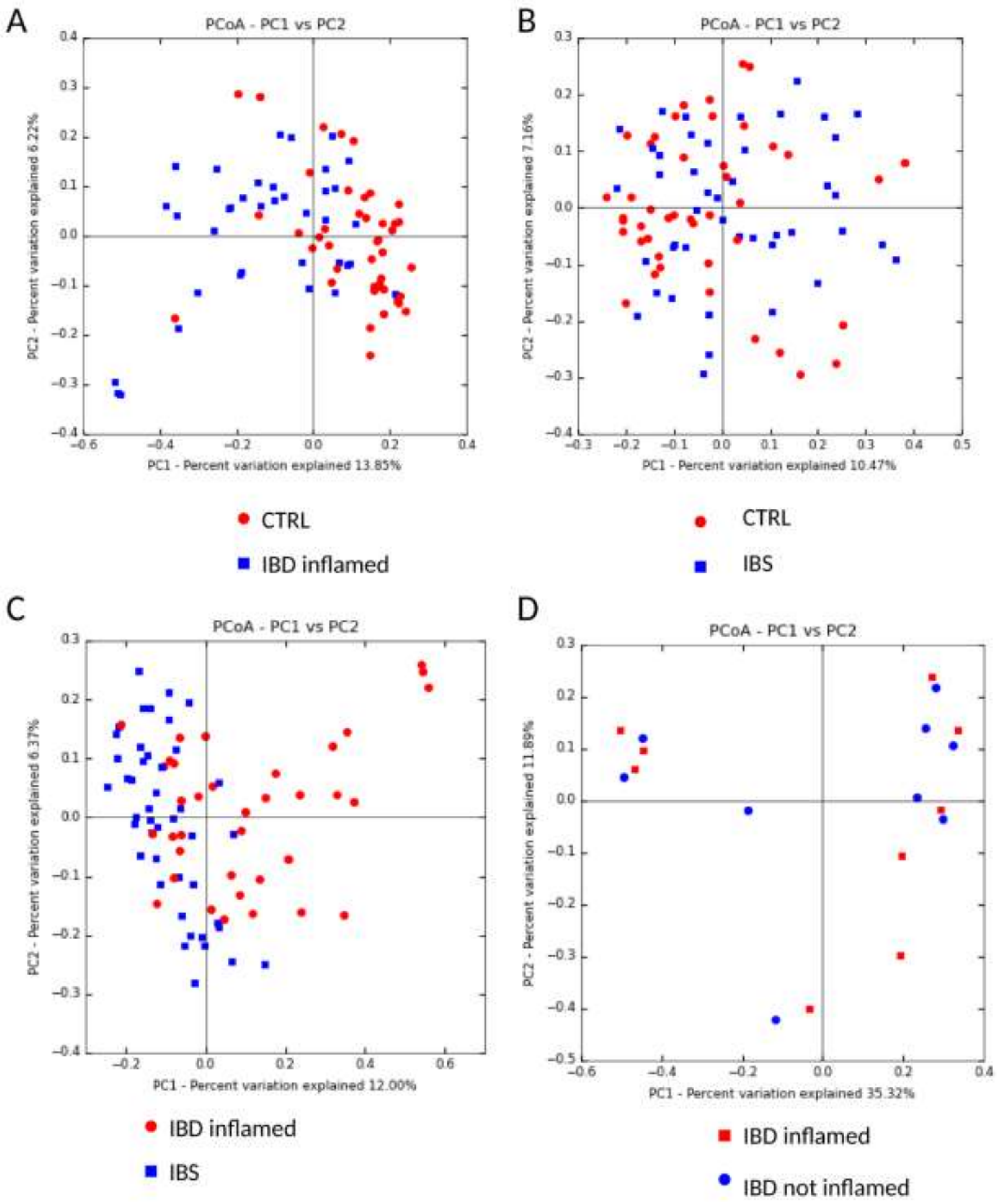

Figure S5. PCoA plot of mucosal samples of IBD and CTRL groups (A), IBS and CTRL (B), IBD and IBS (C) and IBD inflamed and IBD not inflamed (D). The plots show the first two principal coordinates (axes) for PCoA using unweighted UniFrac algorithm.

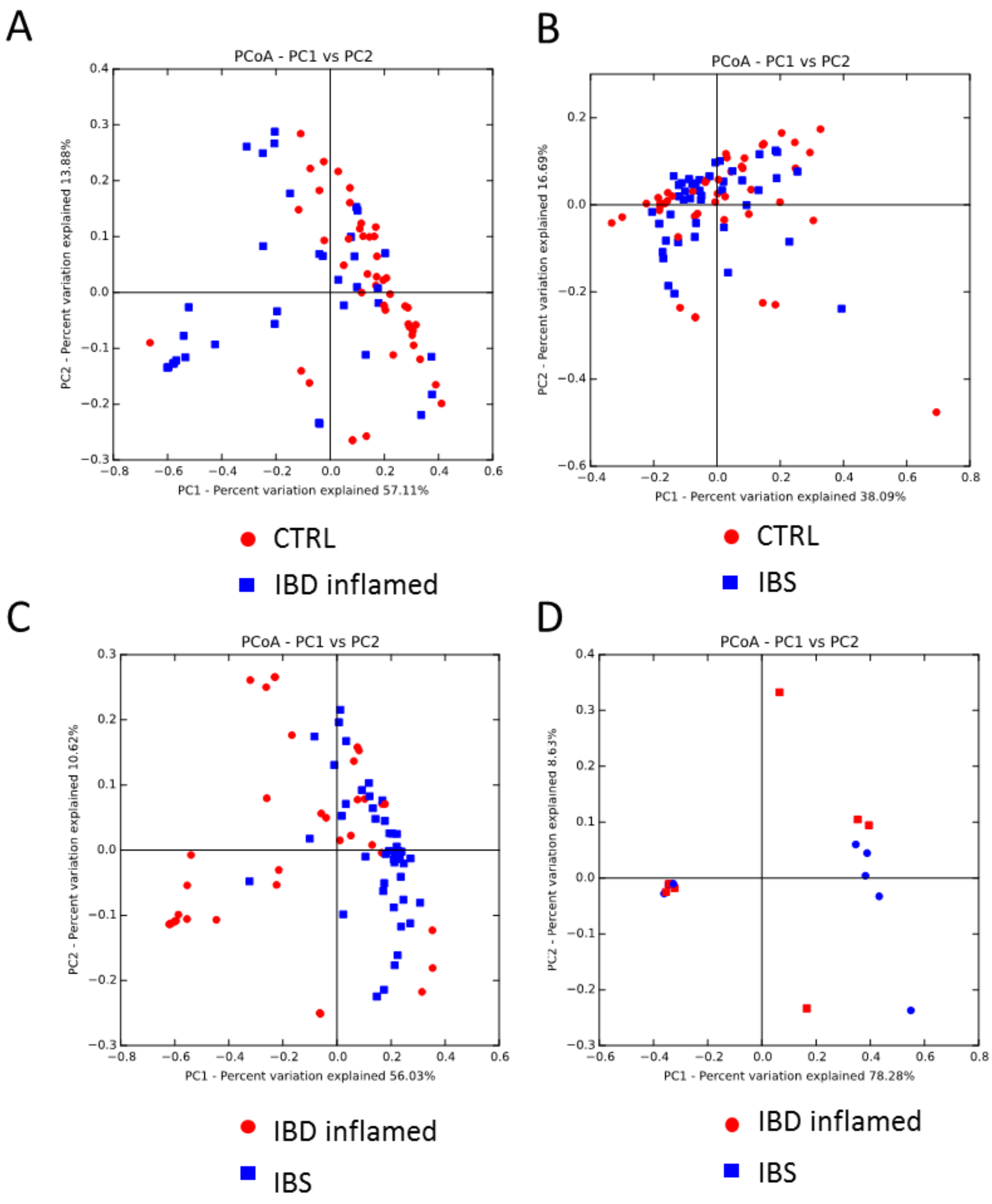

Figure S6. PCoA plot of mucosal samples of IBD and CTRL groups (A), IBS and CTRL (B), IBD and IBS (C) and IBD inflamed and IBD not inflamed (D). The plots show the first two principal coordinates (axes) for PCoA using weighted UniFrac algorithm.

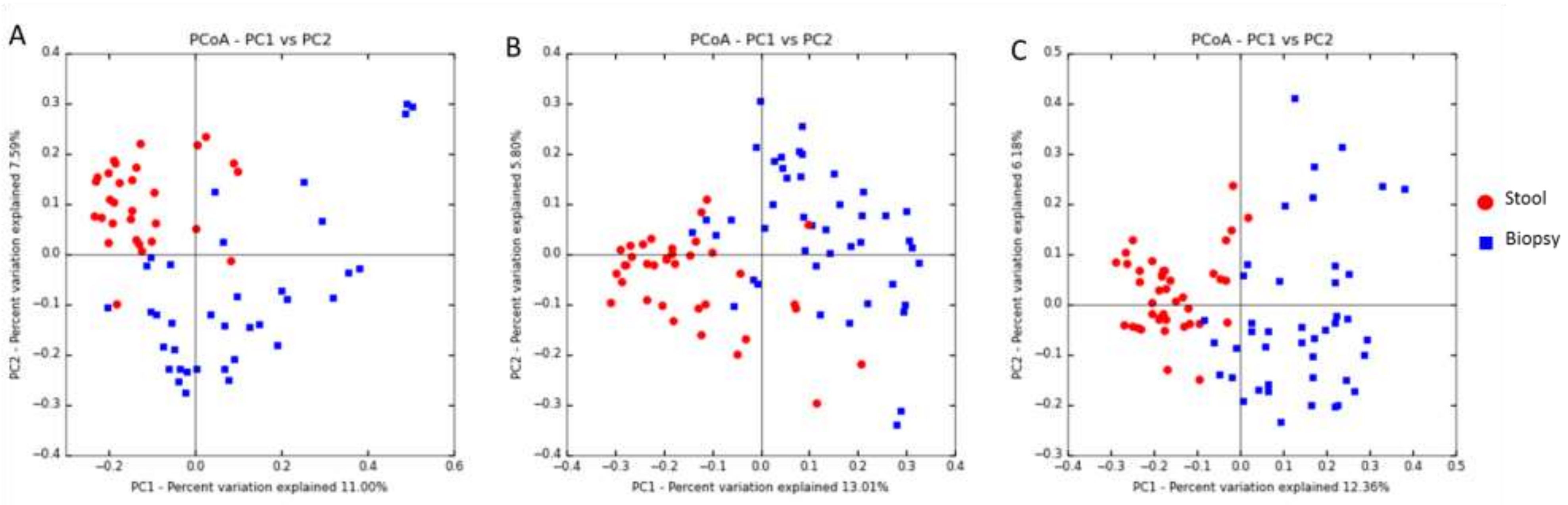

Figure S7. PCoA plot of mucosal and stool samples of IBD (A), IBS (B) and CTRL (C) groups. The plots show the first two principal coordinates (axes) for PCoA using unweighted UniFrac algorithm.

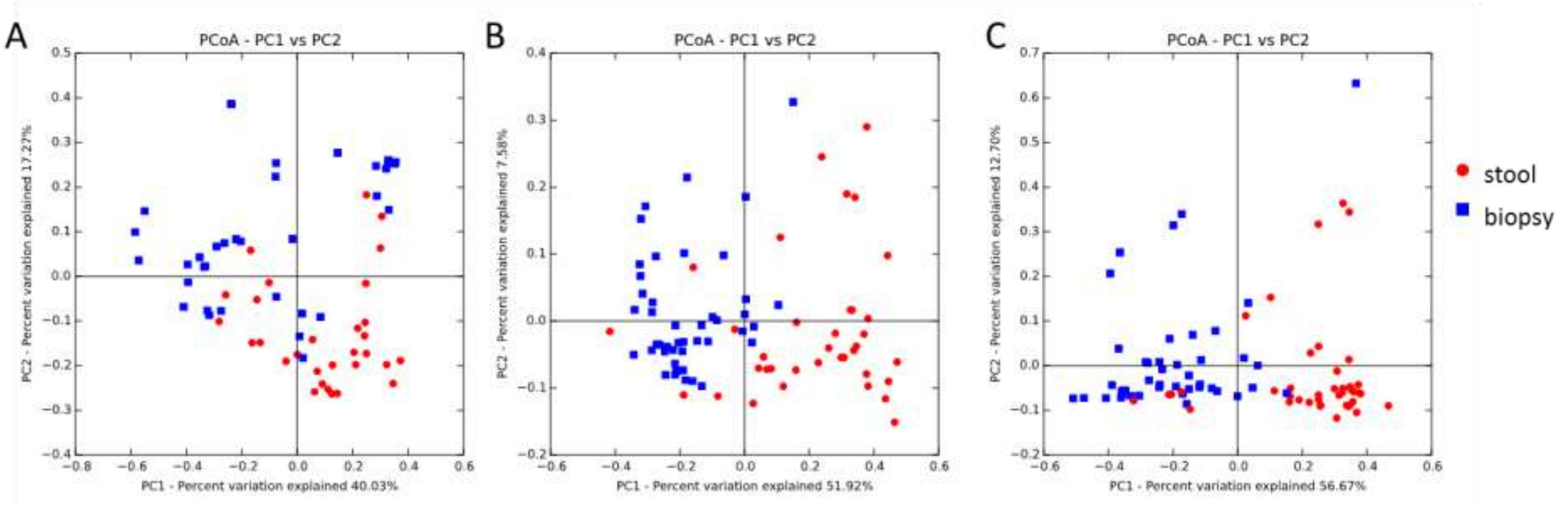

Figure S8. PCoA plot of mucosal and stool samples of IBD (A), IBS (B) and CTRL (C) groups. The plots show the first two principal coordinates (axes) for PCoA using weighted UniFrac algorithm.

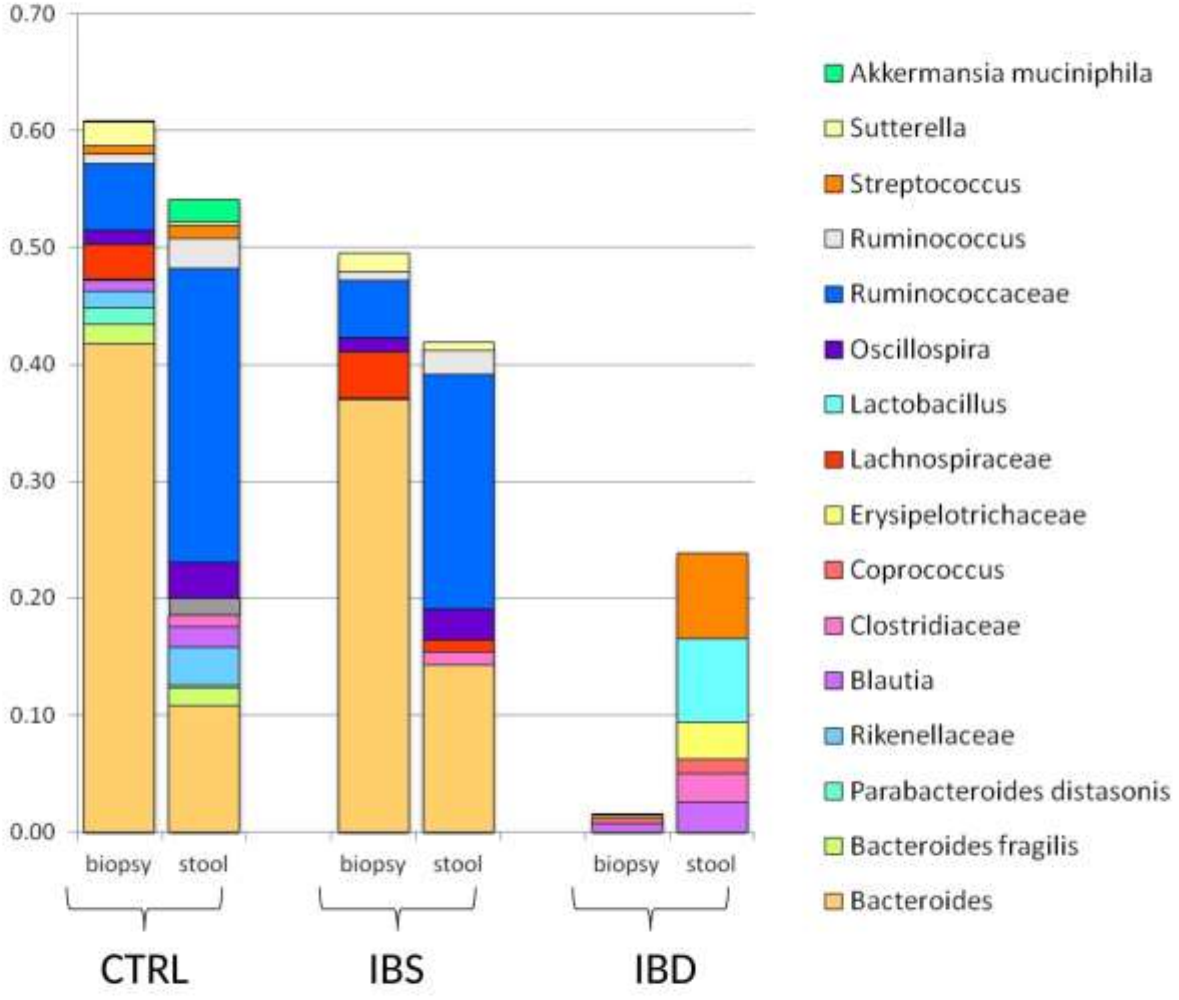

Figure S9. Bar chart reporting Kruskal-Wallis test results on OTUs at families/species distribution of the mucosa versus stool for CTRL, IBS and IBD.

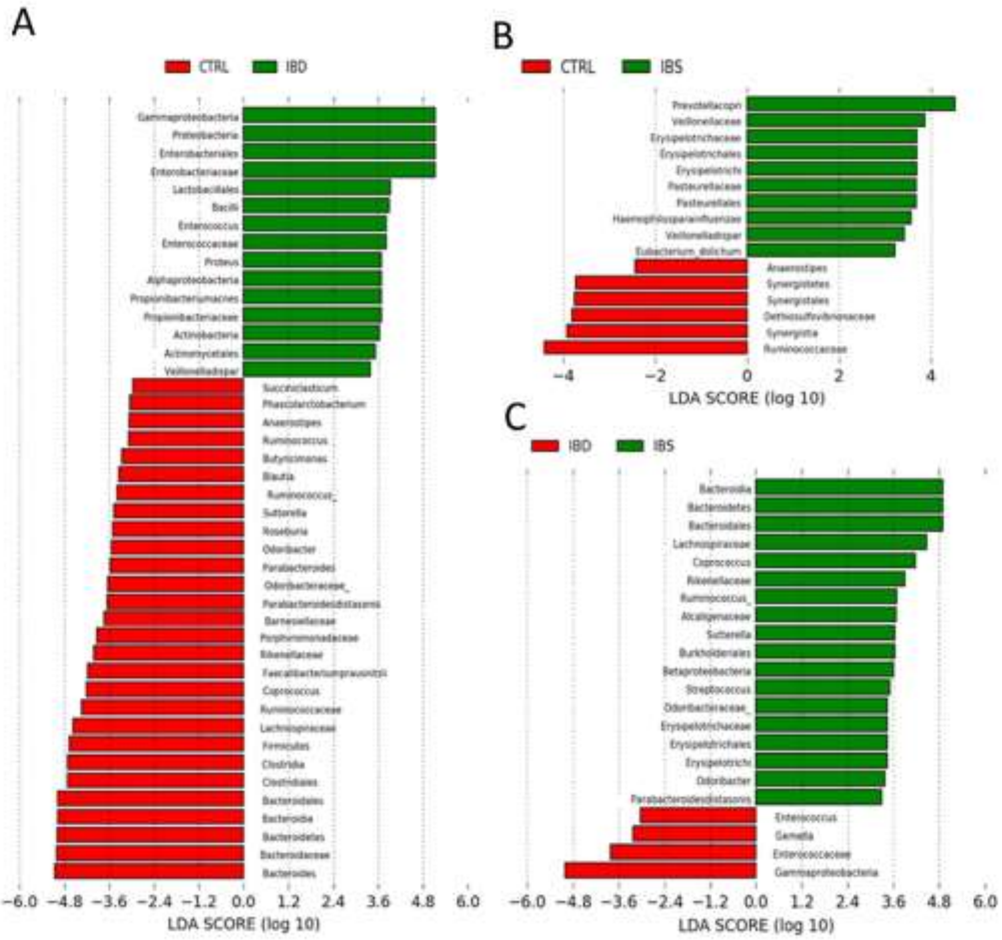

Figure S10. OTU biomarkers associated with biopsy samples from IBS, IBD and CTRL groups. A linear discriminant effect size (LEfSe) analysis was performed (α value = 0.05, logarithmic LDA score threshold = 2.0). In panel A, IBD versus CTRL; B, IBS versus CTRLs; C, IBD versus IBS.

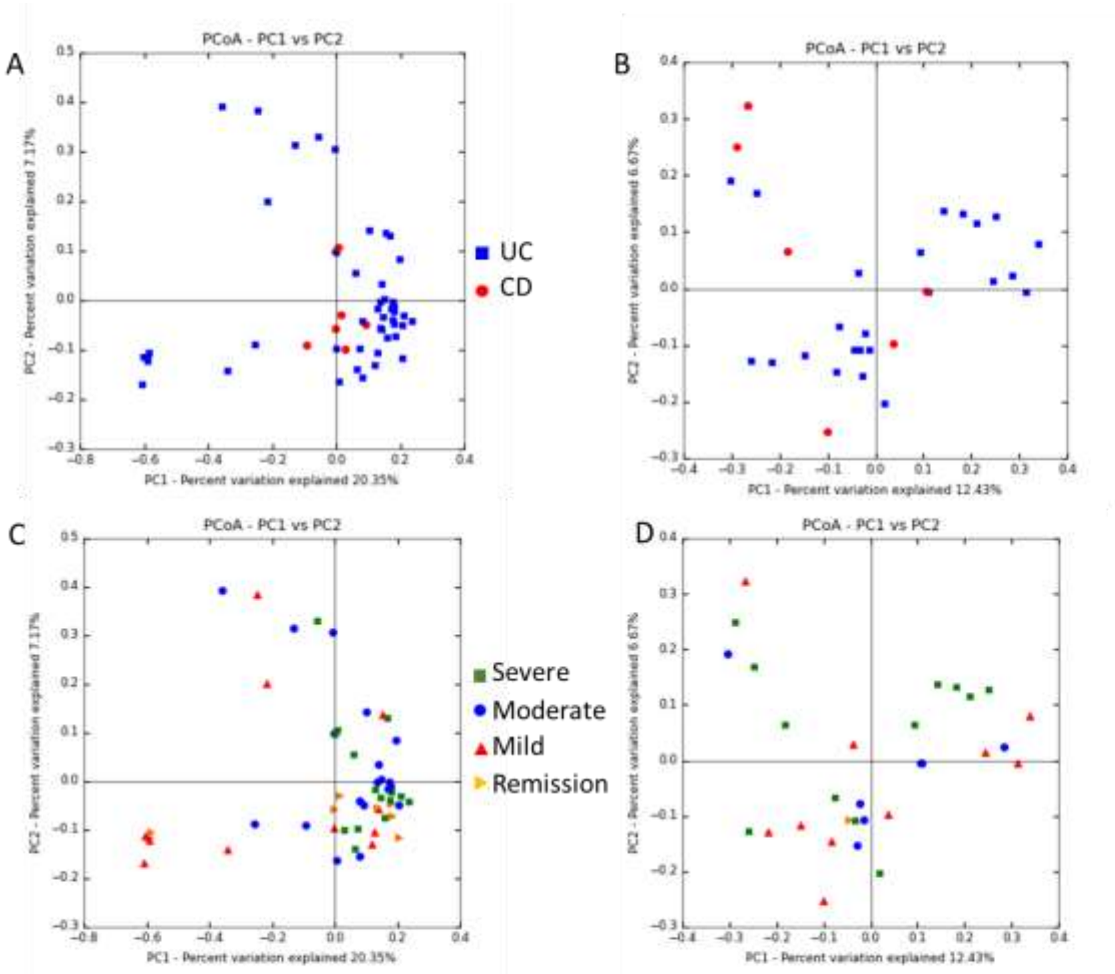

Figure S11. PCoA plot of IBD samples stratified for clinical features. The plots show the first two principal coordinates (axes) for PCoA using unweighted UniFrac algorithm. In panel A and B are reported biopsy and faecal samples, respectively, stratified for UC and CD. In panel C and D are reported biopsy and faecal samples, respectively, stratified for disease activities.

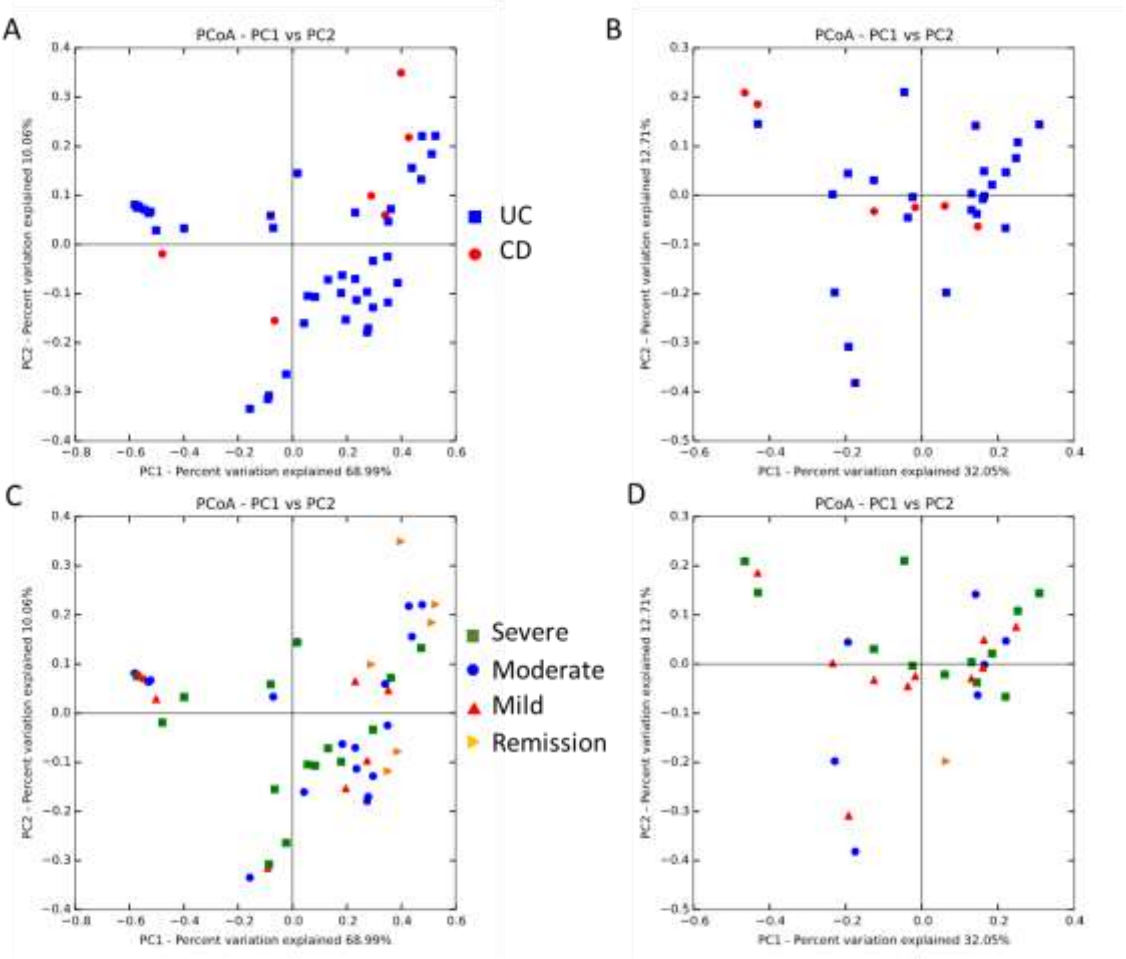

Figure S12. PCoA plot of IBD samples stratified for clinical features. The plots show the first two principal coordinates (axes) for PCoA using weighted UniFrac algorithm. In panel A and B are reported biopsy and faecal samples, respectively, stratified for UC and CD. In panel C and D are reported biopsy and faecal samples, respectively, stratified for disease activities.

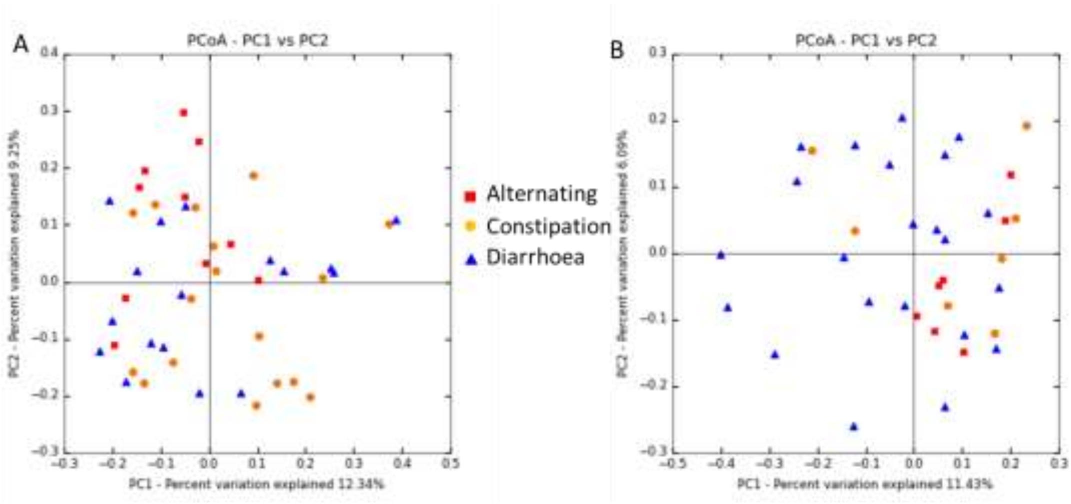

Figure S13. PCoA plot of IBS samples stratified for clinical features. The plots show the first two principal coordinates (axes) for PCoA using unweighted UniFrac algorithm. In panel A and B are reported biopsy and faecal samples, respectively, stratified for predominant bowel habits.

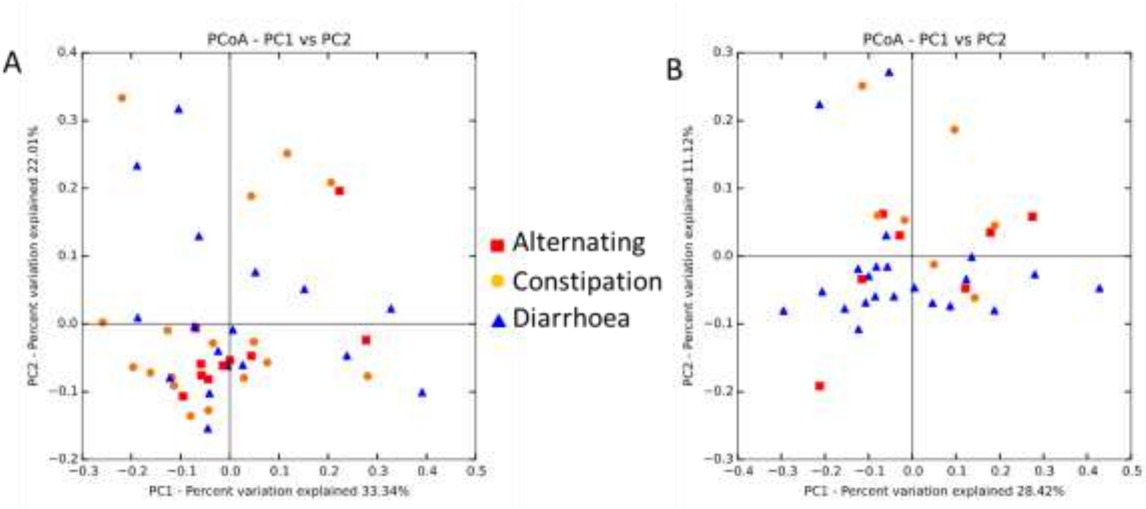

Figure S14. PCoA plot of IBS samples stratified for clinical features. The plots show the first two principal coordinates (axes) for PCoA using weighted UniFrac algorithm. In panel A and B are reported biopsy and faecal samples, respectively, stratified for predominant bowel habits.
